# Artificial intelligence-based evaluation of prognostic benefits from immunotherapy plus targeted therapy with or without radiotherapy or TACE in advanced hepatocellular carcinoma

**DOI:** 10.3389/fonc.2025.1694565

**Published:** 2025-11-05

**Authors:** Yuehong Deng, Shiqin Song, Huarong Zhao, Yuqian Yang, Simin Lu, Xueting Li

**Affiliations:** ^1^ Department of Oncology, Hejiang County People’s Hospital, Luzhou, Sichuan, China; ^2^ Department of Oncology, Chongqing Hygeia Hospital, Chongqing, China; ^3^ Department of Oncology, Luzhou People’s Hospital, Luzhou, China; ^4^ Department of Oncology, 363 Hospital, Chengdu, China

**Keywords:** artificial intelligence, immunotherapy, targeted therapy, radiotherapy, TACE, hepatocellular carcinoma

## Abstract

**Background:**

Hepatocellular carcinoma (HCC) remains a leading cause of cancer-related mortality worldwide, and the prognosis of advanced disease is still poor. Immunotherapy plus targeted therapy has reshaped systemic treatment; however, the overall efficacy is limited. Increasing evidence suggests that combining systemic therapy with locoregional modalities such as transarterial chemoembolization (TACE) or radiotherapy (RT) may improve survival. Artificial intelligence (AI) offers the potential to refine prognostic prediction and optimize patient selection.

**Methods:**

We retrospectively analyzed 351 patients with unresectable HCC, classified into three groups: immunotherapy plus targeted therapy (P+T, *n* = 89), P+T combined with TACE (*n* = 154), and P+T combined with RT (*n* = 108). Univariable Cox regression identified prognostic factors, which were incorporated into five AI models. Model performance was evaluated using the C-index, Brier score, time-dependent receiver operating characteristics (ROC), decision curve analysis (DCA), and calibration.

**Results:**

The median overall survival (mOS) was 12.8 months in the P+T group, 19.7 months in the TACE group (*p* = 0.011), and 22.3 months in the RT group (*p* = 0.030). Among the five AI models, random survival forest (RSF) showed the best performance (C-index = 0.731) with favorable calibration. In the time-dependent ROC analysis, the RSF model achieved area under the curve (AUC) values of 0.844, 0.824, and 0.806 for the prediction of 6-, 12-, and 24-month survival, respectively. DCA indicated a higher net clinical benefit with the RSF model, and the calibration plots showed good agreement between the predicted and the observed survival.

**Conclusion:**

Immunotherapy plus targeted therapy combined with TACE or RT significantly improved survival in advanced HCC compared with systemic therapy alone. RSF provided superior predictive performance and identified critical prognostic variables, supporting AI-assisted approaches as valuable tools for individualized risk stratification and treatment optimization in advanced HCC.

## Introduction

Hepatocellular carcinoma (HCC) is one of the most common malignancies worldwide and ranks among the leading causes of cancer-related mortality, particularly in Asia (1). Despite advances in screening and early detection, the majority of patients are still diagnosed at an advanced stage, and the prognosis remains dismal (2, 3). The median overall survival (mOS) of patients with advanced HCC seldom exceeds 1 year with conventional therapies, highlighting the urgent need for more effective treatment strategies (4).

In recent years, programmed cell death 1 (PD-1) inhibitors combined with molecular targeted agents have reshaped systemic therapy for HCC (5, 6). While these regimens have shown promise, the overall response rate and the durability of the benefit remain unsatisfactory. To enhance treatment efficacy, growing evidence supports combining systemic therapy with locoregional modalities. Among these, transarterial chemoembolization (TACE) and radiotherapy (RT) are the most commonly applied in advanced HCC. Studies suggest that triple therapy—immunotherapy plus targeted therapy together with TACE or RT—may provide superior survival outcomes compared with systemic therapy alone (7, 8).

However, not all patients experience meaningful benefits from these treatment strategies. The high heterogeneity of HCC makes it challenging to identify optimal candidates for immunotherapy, targeted therapy, or their combination with local treatments (9). Artificial intelligence (AI) has emerged as a promising approach to address this issue (10). By integrating diverse data sources—clinical information, imaging biomarkers, and treatment variables—AI-based models can capture complex, nonlinear associations beyond the capacity of conventional statistical tools, thus improving the prediction of treatment benefits (11–13).

Building on this rationale, we designed the present study to explore whether AI can improve prognostic evaluation in advanced HCC treated with immunotherapy plus targeted therapy, with or without RT or TACE. By developing AI-assisted models, we aimed to refine risk stratification, identify patients most likely to benefit from combination strategies, and ultimately provide evidence to guide individualized therapeutic decision-making in clinical practice.

## Materials and methods

### Patient screening and selection

A total of 351 patients with unresectable HCC were retrospectively enrolled from three hospitals in China. Patients were classified into three groups: the PD-1 inhibitor plus targeted therapy group (P+T group, *n* = 89), the PD-1 inhibitor plus targeted therapy combined with TACE group (TACE group, *n* = 154), and the PD-1 inhibitor plus targeted therapy combined with RT group (RT group, *n* = 108).

Participants were selected based on the following inclusion criteria: 1) a clinically or pathologically confirmed diagnosis of HCC; 2) BCLC Barcelona Clinic Liver Cancer (BCLC) stage B/C; 3) Child–Pugh A/B; 4) receipt of P+T; and 5) a subset of patients additionally receiving TACE or RT. The exclusion criteria were as follows: 1) Child–Pugh C; 2) contraindications to TACE or RT; 3) hepatic encephalopathy or refractory ascites; and 4) incomplete clinical data.

This study complied with the Declaration of Helsinki and was approved by the Ethics Committee of Luzhou People’s Hospital. Owing to its retrospective nature, informed consent was waived.

### Treatment

PD-1 inhibitors (such as camrelizumab, tislelizumab, and sintilimab) and targeted agents (i.e., sorafenib or lenvatinib) were administered. In this retrospective study, the decision for patients to receive additional TACE or RT was made collaboratively by the treating physicians and the patients. The considerations included anticipated treatment efficacy, potential toxicities, economic burden, and patient preferences. Written informed consent was obtained from all patients before therapy initiation.

Overall survival (OS) was defined as the time from the start of treatment to death from any cause or the last follow-up.

### AI modeling

Patients were randomly divided into a training set and a validation set at a ratio of 6:4. In the training set, univariate Cox regression was first performed to identify statistically significant prognostic variables for inclusion in the subsequent machine learning models. While univariate Cox regression captures linear relationships, it may overlook complex, nonlinear interactions. To address this, several machine learning models were used, including random survival forest (RSF), least absolute shrinkage and selection operator (LASSO), gradient boosting machine (GBM), decision tree (DT), and support vector machine (SVM), in order to capture nonlinear associations and enhance the predictive accuracy. The model with the highest concordance index (C-index) in the training set was selected. In the validation set, model performance was evaluated using the Brier score, time-dependent receiver operating characteristic (ROC) curves, decision curve analysis (DCA), and calibration plots.

To enhance interpretability, variable importance plots and partial dependence plots (PDPs) were generated.

### Statistical analysis

Differences between categorical variables were compared using the chi-square test, while continuous variables were analyzed with the Student’s *t*-test or the Mann–Whitney *U* test, depending on data distribution. Survival curves were estimated using the Kaplan–Meier method, and group differences were assessed with the log-rank test. All statistical analyses were performed with R software (version 4.4.3), and a two-sided *p*-value <0.05 was considered statistically significant.

## Results

### Clinical features and survival outcomes

A total of 351 patients with unresectable HCC were included, with 89 in the P+T group, 154 in the TACE group, and 108 in the RT group. The majority of the patients were men (83.1%–89.8%) and positive for hepatitis B virus (HBV) (63.0%–74.7%). The majority had Child–Pugh A liver function (71.3%–75.3%) and advanced BCLC stage C disease (79.2%–93.5%). Tumor burden was high, with the majority of patients presenting with two or more nodules (70.4%–85.1%) and a large tumor size (≥5 cm in the majority). Portal vein tumor thrombus (PVTT) was present in approximately 55.8%–67.6% of patients, while extrahepatic spread (M1) was observed in 27.8%–40.4%. Compared with the P+T group, no significant differences were observed in the baseline characteristics between the TACE group and the RT group, including demographic factors, liver function, tumor burden, disease stage, and laboratory parameters (**Table 1**).

**Table 1:** Baseline characteristics of the patients in the P+T, TACE, and RT groups.

	P+t	TACE	*P* ^a^	RT	*P*b
Patients, *n*	89	154		108	
Age (years), mean ± SD	54.2 ± 11.2	52.2 ± 10.4	0.175	53.8 ± 11.5	0.823
Sex			0.524		0.244
Women	15 (16.9%)	20 (13.0%)		11 (10.2%)	
Men	74 (83.1%)	134 (87.0%)		97 (89.8%)	
HBV			0.212		0.737
No	30 (33.7%)	39 (25.3%)		40 (37.0%)	
Yes	59 (66.3%)	115 (74.7%)		68 (63.0%)	
Child–Pugh			0.665		1.000
A	64 (71.9%)	116 (75.3%)		77 (71.3%)	
B	25 (28.1%)	38 (24.7%)		31 (28.7%)	
AFP			0.317		1.000
<400	46 (51.7%)	68 (44.2%)		55 (50.9%)	
≥400	43 (48.3%)	86 (55.8%)		53 (49.1%)	
BCLC			0.307		0.101
B	13 (14.6%)	32 (20.8%)		7 (6.48%)	
C	76 (85.4%)	122 (79.2%)		101 (93.5%)	
Number			0.507		0.125
1	17 (19.1%)	23 (14.9%)		32 (29.6%)	
≥2	72 (80.9%)	131 (85.1%)		76 (70.4%)	
Size			0.410		0.578
<5	22 (24.7%)	29 (18.8%)		25 (23.1%)	
≥ 5, <10	34 (38.2%)	56 (36.4%)		49 (45.4%)	
≥10	33 (37.1%)	69 (44.8%)		34 (31.5%)	
PVTT			0.549		0.390
No	35 (39.3%)	68 (44.2%)		35 (32.4%)	
Yes	54 (60.7%)	86 (55.8%)		73 (67.6%)	
N			0.797		0.603
No	37 (41.6%)	68 (44.2%)		50 (46.3%)	
Yes	52 (58.4%)	86 (55.8%)		58 (53.7%)	
M			0.051		0.085
No	53 (59.6%)	112 (72.7%)		78 (72.2%)	
Yes	36 (40.4%)	42 (27.3%)		30 (27.8%)	
Leukocyte	6.29 ± 2.27	6.34 ± 2.55	0.862	6.68 ± 2.49	0.250
<4	11 (12.4%)	19 (12.3%)		14 (13.0%)	
≥4	78 (87.6%)	135 (87.7%)		94 (87.0%)	
Platelet	172 ± 94.6	168 ± 89.1	0.717	173 ± 82.1	0.916
<100	22 (24.7%)	33 (21.4%)		20 (18.5%)	
≥100	67 (75.3%)	121 (78.6%)		88 (81.5%)	
ALT	58.6 ± 47.7	62.6 ± 108	0.685	64.5 ± 73.4	0.495
<40	36 (40.4%)	88 (57.1%)		53 (49.1%)	
≥40	53 (59.6%)	66 (42.9%)		55 (50.9%)	
AST	90.2 ± 78.0	78.2 ± 72.0	0.238	78.2 ± 76.8	0.283
<40	23 (25.8%)	44 (28.6%)		35 (32.4%)	
≥40	66 (74.2%)	110 (71.4%)		73 (67.6%)	

*P+T*, PD-1 inhibitors plus targeted therapy; *TACE*, transarterial chemoembolization; *RT*, radiotherapy; *HBV*, hepatitis B virus; *AFP*, alpha-fetoprotein; *BCLC*, Barcelona Clinic Liver Cancer; *PVTT*, portal vein tumor thrombosis; *N*, lymph node involvement; *M*, metastasis; *ALT*, alanine aminotransferase; *AST*, aspartate aminotransferase.

a P+T *vs*. TACE.

b P+T *vs*. RT.

**Figure 1:**
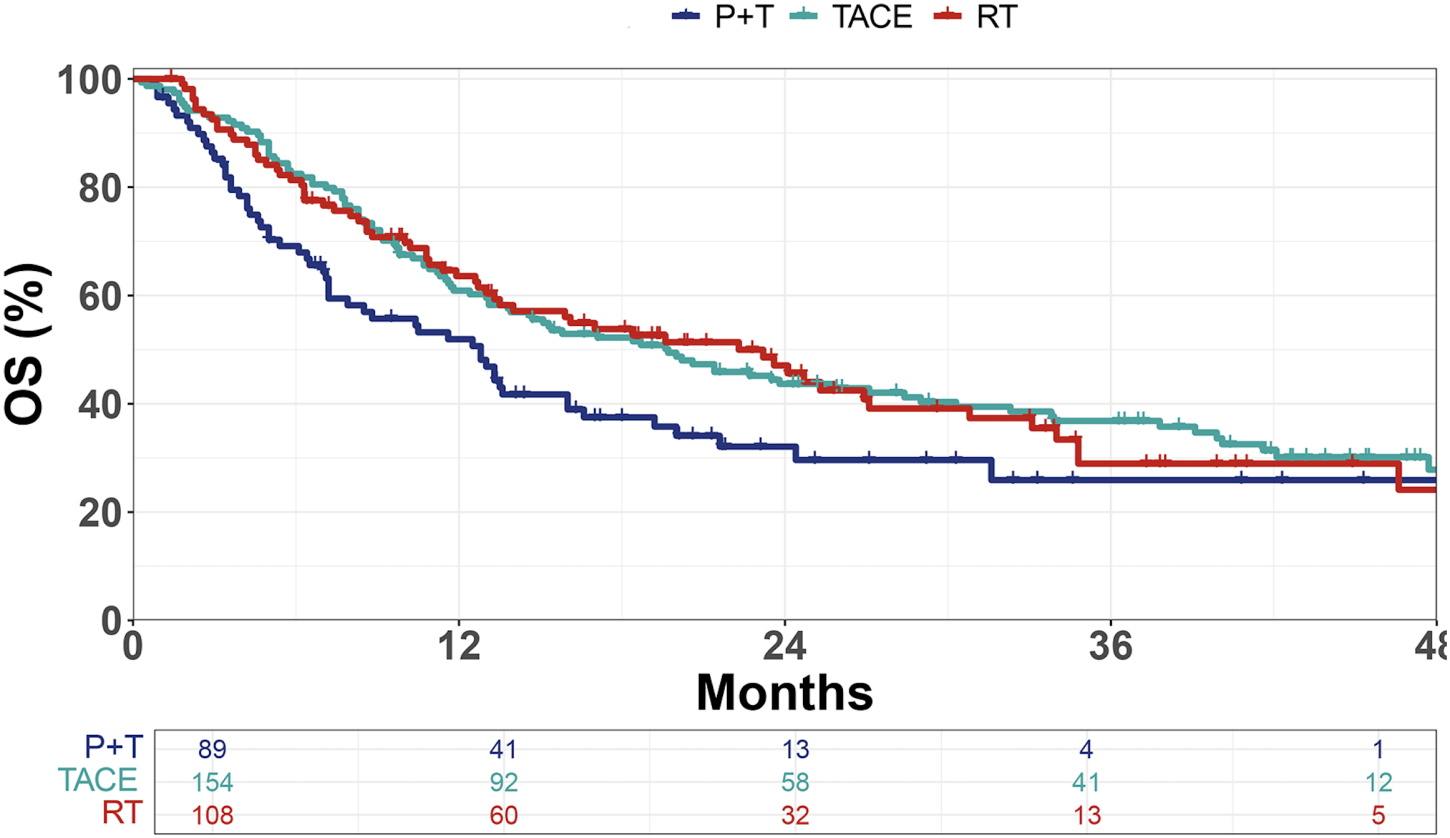
Kaplan–Meier curves of overall survival (OS) in patients treated with PD-1 inhibitors plus targeted therapy (P+T), transarterial chemoembolization (TACE), or radiotherapy (RT). *OS*, overall survival.

Compared with the P+T group (mOS = 12.8 months), both the TACE group (mOS = 19.7 months, *p* = 0.011) and the RT group (mOS = 22.3 months, *p* = 0.030) demonstrated significant survival benefits (**Figure 1**).

### Prognostic factor selection

Univariable Cox analysis demonstrated that OS was significantly associated with age, the Child–Pugh score, the alpha-fetoprotein (AFP) level, the BCLC stage, the tumor size, PVTT, metastasis, aspartate aminotransferase (AST), and treatment (**Table 2**).

**Table 2:** Univariable Cox for overall survival.

	HR	Univariable	*p*
Age (years)	0.99	0.97–1.00	0.033
Sex (men/women)	0.83	0.57–1.20	0.322
HBV (positive/negative)	1.33	0.99–1.79	0.062
Child (B/A)	1.70	1.28–2.27	<0.001
AFP (≥400/<400 ng/ml)	1.58	1.21–2.07	<0.001
BCLC (C/B)	2.60	1.64–4.12	<0.001
Number (≥2/<2)	1.27	0.90–1.79	0.178
Size			
<5	Reference		
≥ 5, <10	1.25	0.85–1.83	0.261
≥10	1.88	1.30–2.74	<0.001
PVTT (positive/negative)	1.85	1.39–2.46	<0.001
N (positive/negative)	1.24	0.95–1.62	0.115
M (positive/negative)	1.56	1.18–2.06	0.002
Leukocyte	1.01	0.96–1.07	0.589
Platelet	1.00	1.00–1.00	0.675
ALT	1.00	1.00–1.00	0.107
AST	1.00	1.00–1.00	0.002
Treatment			
P+T	Reference		
TACE	0.65	0.47–0.91	0.011
RT	0.67	0.47–0.96	0.03

*P+T*, PD-1 inhibitors plus targeted therapy; *TACE*, transarterial chemoembolization; *RT*, radiotherapy; *HBV*, hepatitis B virus; *AFP*, alpha-fetoprotein; *BCLC*, Barcelona Clinic Liver Cancer; *PVTT*, portal vein tumor thrombosis; *N*, lymph node involvement; *M*, metastasis; *ALT*, alanine aminotransferase; *AST*, aspartate aminotransferase

### AI model

A total of 351 patients were allocated into a training cohort (*n* = 210) and a validation cohort (*n* = 141) at a 6:4 ratio. The baseline characteristics were comparable between the two cohorts (**Supplementary Table S1**).

**Figure 2:**
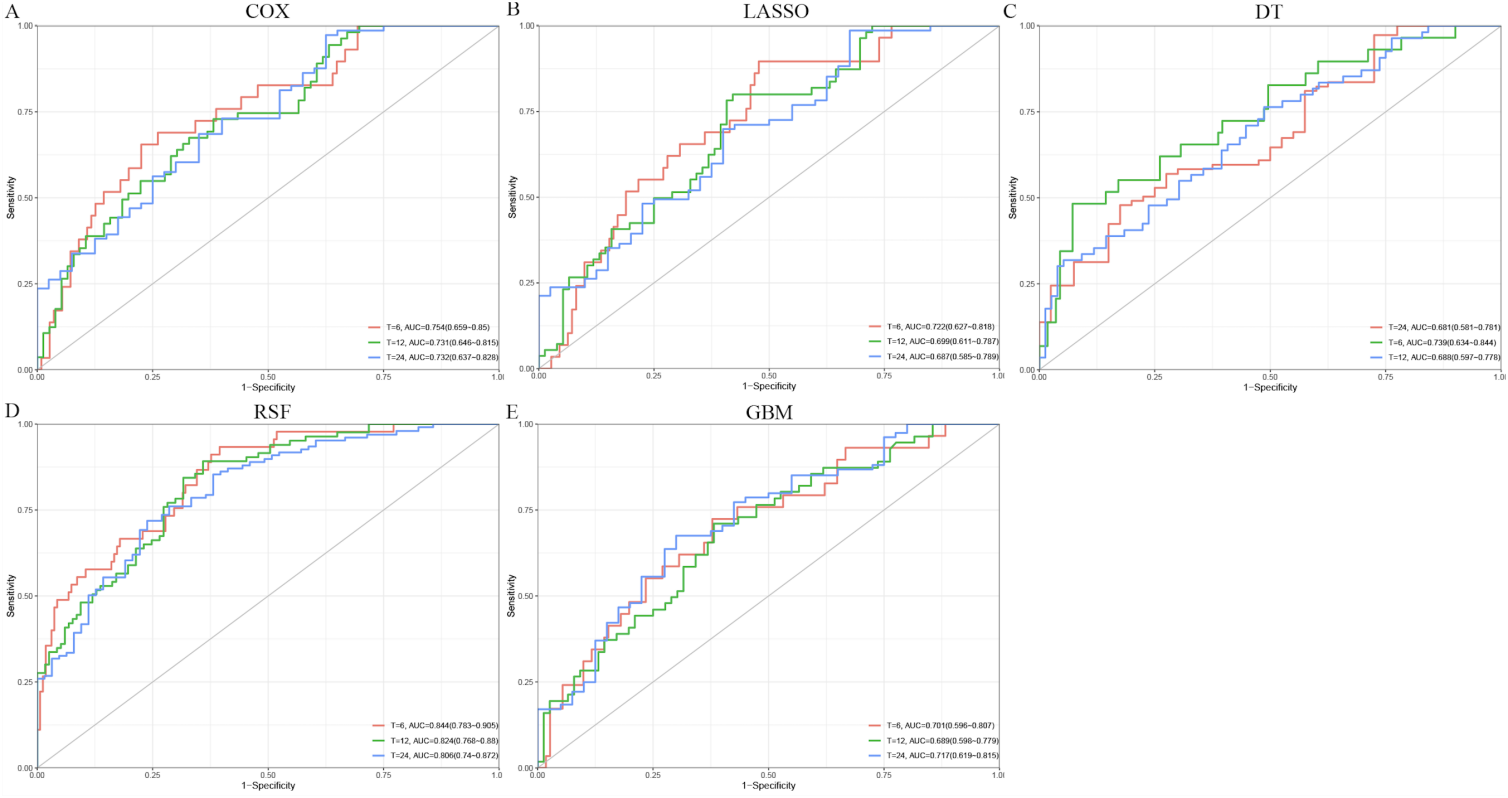
Receiver operating characteristic (ROC) curves for predicting overall survival using different models. **(A)** Cox. **(B)** Least absolute shrinkage and selection operator (LASSO). **(C)** Decision tree (DT). **(D)** Random survival forest (RSF). **(E)** Gradient boosting machine (GBM).

In the training set, the risk factors identified by univariable Cox regression were incorporated into the multivariable Cox, LASSO, DT, RSF, and GBM models. Among these, the RSF model achieved the highest concordance index (C-index = 0.731). In the validation set, the RSF model showed favorable calibration, with Brier scores of 0.144, 0.215, and 0.218 at 6, 12, and 24 months, respectively (**Table 3**). For the time-dependent ROC analysis, the area under the curve (AUC) values at 6, 12, and 24 months were, respectively, 0.754, 0.731, and 0.732 for the Cox model (**Figure 2A**); 0.722, 0.699, and 0.687 for LASSO (**Figure 2B**); 0.681, 0.739, and 0.688 for DT (**Figure 2C**); 0.844, 0.824, and 0.806 for RSF (**Figure 2D**); and 0.701, 0.689, and 0.717 for GBM (**Figure 2E**). The DCA showed greater net clinical benefits than the treat-all or treat-none strategies at 6 months (**Supplementary Figure 1A**), 12 months (**Supplementary Figure 1B**), and 24 months (**Supplementary Figure 1C**). The calibration curves (**Supplementary Figure 1D**) indicated good consistency between the predicted and the observed survival at each time point.

**Table 3:** Performance comparison of the different models for survival prediction.

Model	C-index	Brier (6 months)	Brier (12 months)	Brier (24 months)	ROC (6 months)	ROC (12 months)	ROC (24 months)
Cox	0.664	0.146	0.206	0.205	0.754	0.731	0.732
LASSO	0.667	0.154	0.222	0.224	0.722	0.699	0.687
DT	0.647	0.144	0.215	0.218	0.681	0.739	0.688
RSF	0.731	0.134	0.185	0.189	0.844	0.824	0.806
GBM	0.694	0.169	0.272	0.295	0.701	0.689	0.717

*ROC*, receiver operating characteristics; *LASSO*, least absolute shrinkage and selection operator; *DT*, decision tree; *RSF*, random survival forest; *GBM*, gradient boosting machine

**Figure 3:**
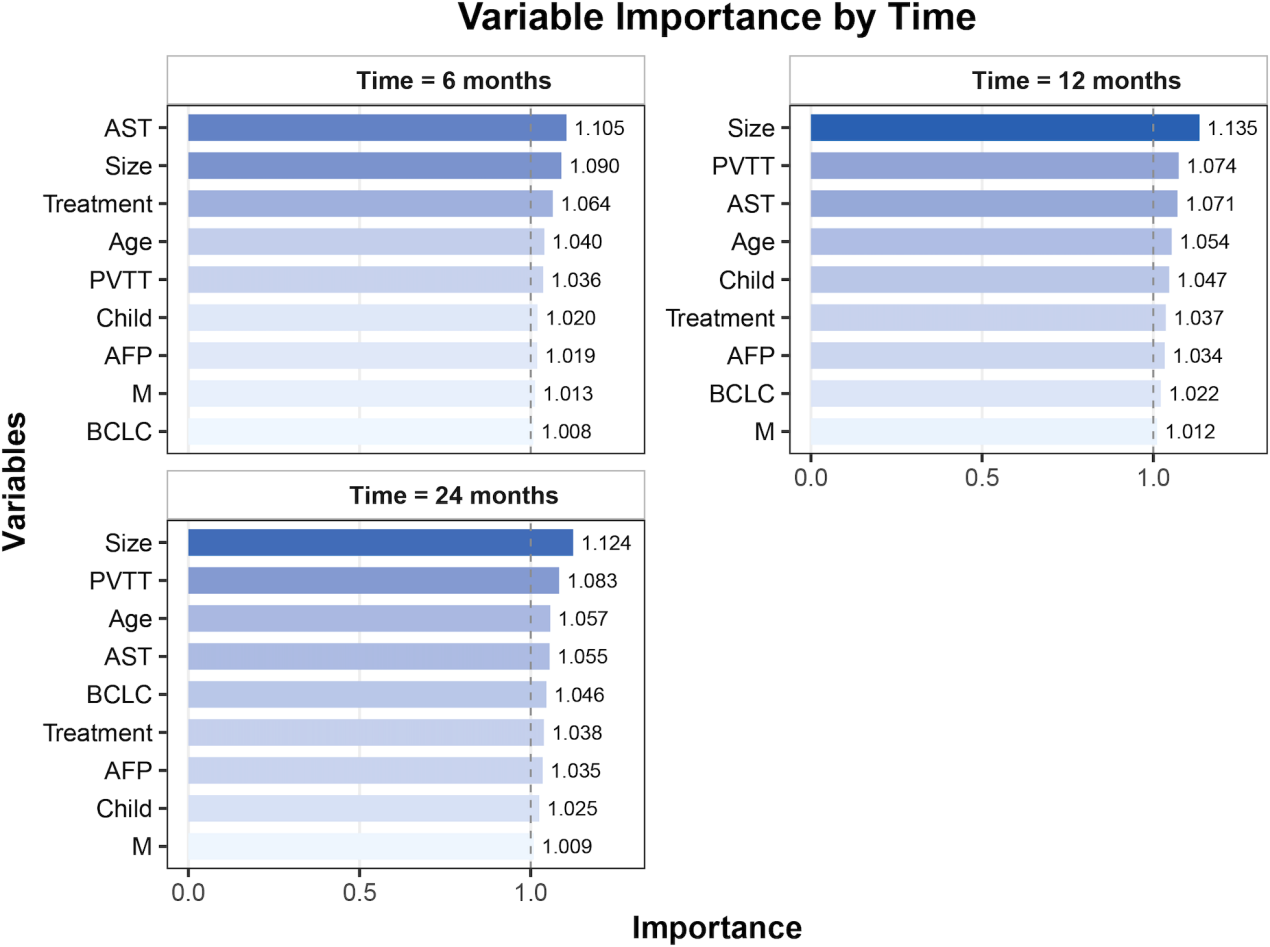
Variable importance for overall survival prediction at different time points (6, 12, and 24 months). *AST*, aspartate aminotransferase; *PVTT*, portal vein tumor thrombosis; *AFP*, alpha-fetoprotein; *BCLC*, Barcelona Clinic Liver Cancer; *M*, metastasis.

**Figure 4:**
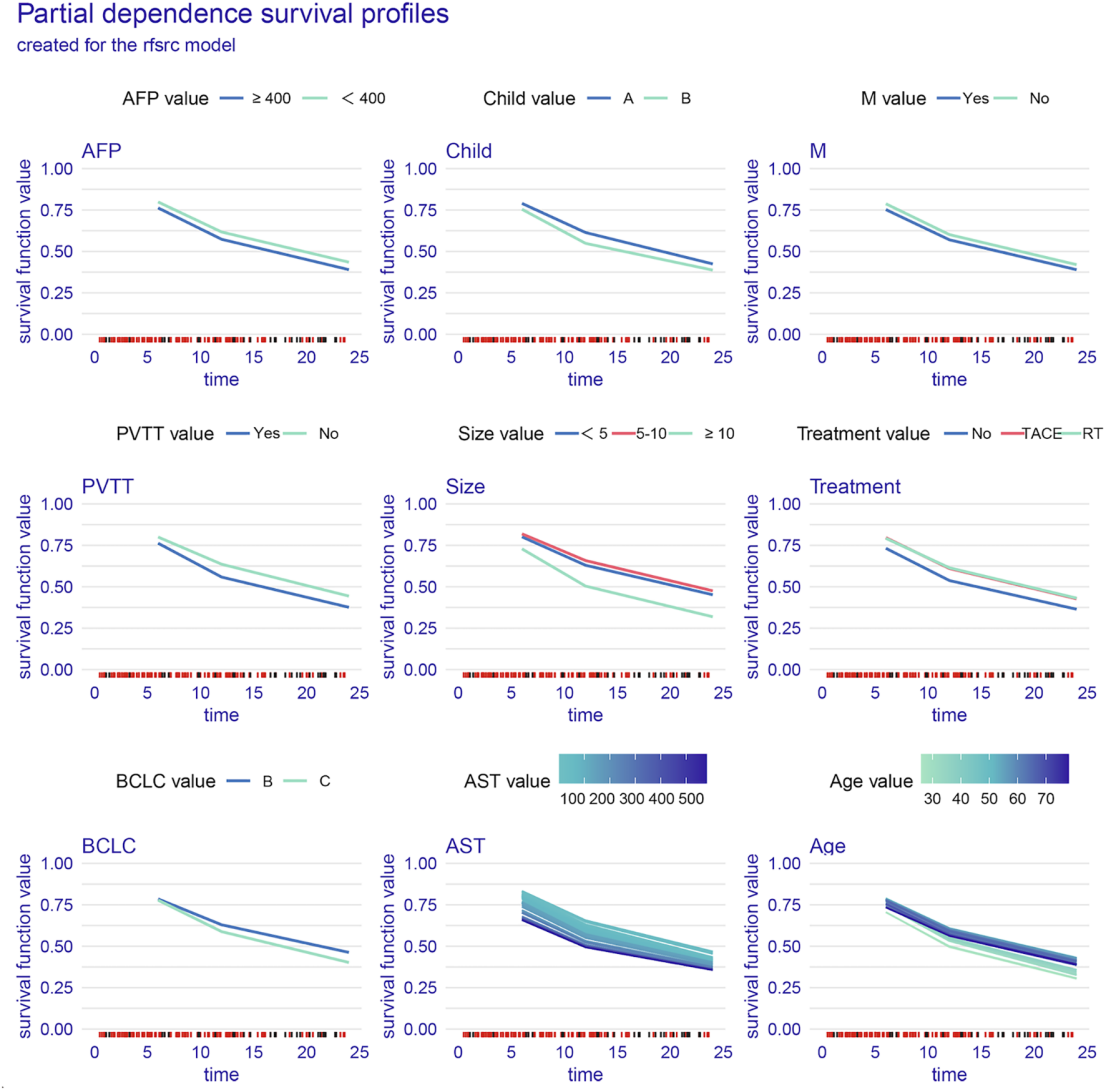
Partial dependence survival profiles for key clinical variables. *AST*, aspartate aminotransferase; *PVTT*, portal vein tumor thrombosis; *AFP*, alpha-fetoprotein; *BCLC*, Barcelona Clinic Liver Cancer; *M*, metastasis.

Variable importance analysis of the RSF model revealed dynamic prognostic patterns across different time points. At 6 months, AST, tumor size, and treatment were the top contributors to survival prediction. By 12 months, tumor size, PVTT, and AST became the most influential variables. At 24 months, tumor size and PVTT consistently remained the strongest predictors, followed by age and AST (**Figure 3**). The PDP of the RSF model showed that unfavorable clinical factors, including a high AFP, Child–Pugh B, the presence of PVTT or metastasis, advanced BCLC stage, a large tumor size, and an elevated AST, were consistently associated with poorer survival probabilities. In contrast, patients receiving TACE or RT demonstrated improved survival compared with those on systemic therapy alone (**Figure 4**).

## Discussion

In this retrospective study, we confirmed that, in patients with advanced HCC, immunotherapy plus targeted therapy combined with a locoregional treatment (i.e., TACE or RT) significantly improved survival compared with systemic therapy alone. Specifically, the mOS reached 19.7 months in the TACE group and 22.3 months in the RT group, while it was only 12.8 months in the P+T group. Furthermore, the application of AI-based modeling refined the prognostic evaluation and improved survival prediction, highlighting its value in clinical decision-making.

The survival benefits of triple therapy are supported by several high-impact studies. Llovet et al. (14) reported that the combination of systemic and locoregional therapy improved tumor control and prolonged survival in advanced HCC. McPartlin and Dawson (15) and Tan et al. (16) demonstrated that the addition of locoregional modalities could enhance the efficacy of systemic therapy by promoting antigen release, improving T-cell infiltration, and remodeling the tumor microenvironment. Similarly, Sun et al. (17) emphasized that the integration of local and systemic approaches could overcome resistance mechanisms and synergistically enhance therapeutic efficacy. Together, these findings support the rationale for triple therapy in certain patients.

AI provides unique advantages in prognostic modeling (18). Traditional Cox regression is constrained by linear assumptions and proportional hazards, which may oversimplify relationships in heterogeneous populations. In contrast, AI models can accommodate nonlinear interactions and high-dimensional data (19). In this study, the RSF model outperformed Cox, LASSO, DT, and GBM, achieving the highest C-index (0.731) and demonstrating superior calibration, ROC, and DCA results. As an ensemble learning method, RSF was used in this study due to its capacity to directly handle high-dimensional variables without the need for preselection. RSF, as an ensemble method, performs automatic variable selection during model fitting, thereby identifying the most informative predictors from a large pool of variables. This approach is particularly useful in high-dimensional settings as it reduces overfitting, handles missing data efficiently, and avoids the loss of important nonlinear predictors that may be excluded by traditional variable selection methods (20–23).

While the RSF model demonstrates strong predictive performance, its practical application in clinical decision-making remains underexplored. The model can be used to stratify patients based on predicted survival outcomes, enabling clinicians to identify those most likely to benefit from specific treatments. For instance, patients predicted to have a poor prognosis may be prioritized for more aggressive treatments or closer monitoring, while those with a better prognosis may be considered for less intensive therapies, thus optimizing resource allocation and minimizing unnecessary toxicity. Furthermore, the ability of the RSF model to integrate complex, high-dimensional data from clinical variables, treatments, and biomarkers makes it a valuable tool for personalized treatment strategies, aligning with the goals of precision medicine.

The variable importance and partial dependence analyses revealed that tumor size, PVTT, and AST were consistently the most influential prognostic factors at 6, 12, and 24 months. These variables are well-established prognostic factors in clinical practice. A larger tumor size and the presence of PVTT indicate a higher tumor burden and a more advanced disease, both of which are known to be associated with poor prognosis (24, 25). Similarly, an elevated AST level reflects liver function impairment, which is crucial in predicting patient outcomes in HCC, as liver dysfunction is a key determinant of treatment response and survival (26). The importance of these variables aligns with current clinical knowledge, confirming their role in guiding treatment decisions for patients with HCC. Our findings suggest that these factors, when considered in combination, can provide a more robust prediction of survival outcomes and help personalize treatment strategies (27). Patients with large tumors, with PVTT, or with an elevated AST exhibited significantly worse outcomes, whereas those treated with TACE or RT demonstrated persistently improved survival (28–30). This reinforces the clinical value of integrating locoregional and systemic therapies in the management of advanced HCC.

The clinical implications of these findings are substantial. Advanced HCC is highly heterogeneous, and uniform treatment strategies may not be optimal. AI-assisted prognostic models such as RSF provide data-driven tools to stratify patients by risk and identify those most likely to benefit from triple therapy. This individualized approach facilitates tailored treatment decisions, improves cost-effectiveness, and reduces unnecessary toxicity for patients unlikely to respond, thereby advancing precision oncology in HCC.

However, several limitations must be acknowledged. Firstly, the retrospective nature of the study carries inherent risks of selection bias despite the balanced baseline features across groups. Secondly, heterogeneity in the treatment strategies, including the RT dose, the TACE protocols, and the selection of PD-1 inhibitors or targeted agents, may have influenced the outcomes. Thirdly, the study population was derived from three centers in China, potentially limiting generalizability. Finally, external validation in larger, prospective, and ethnically diverse cohorts, as well as the integration of radiomics, genomics, and immune profiling, will be necessary to further enhance the predictive performance of AI models.

In conclusion, this study demonstrated that immunotherapy plus targeted therapy combined with TACE or RT significantly prolonged survival in patients with advanced HCC compared with systemic therapy alone. The RSF model exhibited superior predictive performance and identified key prognostic variables, providing a robust AI-based framework for individualized prognostic evaluation. These findings underscore the potential of integrating AI with multimodal treatment strategies to refine risk stratification and optimize therapeutic decision-making in advanced HCC.

## Data Availability

The raw data supporting the conclusions of this article will be made available by the authors, without undue reservation.
