# Supplementary material for: Artificial intelligence-based evaluation of prognostic benefits from immunotherapy plus targeted therapy with or without radiotherapy or TACE in advanced hepatocellular carcinoma
Source: Front Oncol. 2025 Nov 5;15:1694565. doi: 10.3389/fonc.2025.1694565 (PMC12626802; doi:10.3389/fonc.2025.1694565)

| **Supplementary Table1.** Baseline Characteristics of Patients in the Overall, Test, and Training Cohorts | | | | |
| --- | --- | --- | --- | --- |
|  | ALL | Test | Train | P |
| Patients | 351 | 141 | 210 |  |
| Age | 53.2 ± 11.0 | 53.4 ± 11.2 | 53.1 ± 10.8 | 0.803 |
| Child |  |  |  | 1.000 |
| A | 257 (73.2%) | 103 (73.0%) | 154 (73.3%) |  |
| B | 94 (26.8%) | 38 (27.0%) | 56 (26.7%) |  |
| AFP |  |  |  | 0.460 |
| ＜ 400 | 169 (48.1%) | 64 (45.4%) | 105 (50.0%) |  |
| ≥ 400 | 182 (51.9%) | 77 (54.6%) | 105 (50.0%) |  |
| BCLC |  |  |  | 0.621 |
| B | 52 (14.8%) | 23 (16.3%) | 29 (13.8%) |  |
| C | 299 (85.2%) | 118 (83.7%) | 181 (86.2%) |  |
| Size |  |  |  | 0.622 |
| ＜ 5 | 76 (21.7%) | 28 (19.9%) | 48 (22.9%) |  |
| ≥ 5,＜ 10 | 139 (39.6%) | 60 (42.6%) | 79 (37.6%) |  |
| ≥ 10 | 136 (38.7%) | 53 (37.6%) | 83 (39.5%) |  |
| PVTT |  |  |  | 0.835 |
| No | 138 (39.3%) | 54 (38.3%) | 84 (40.0%) |  |
| Yes | 213 (60.7%) | 87 (61.7%) | 126 (60.0%) |  |
| M |  |  |  | 1.000 |
| No | 243 (69.2%) | 98 (69.5%) | 145 (69.0%) |  |
| Yes | 108 (30.8%) | 43 (30.5%) | 65 (31.0%) |  |
| AST | 81.3 ± 75.0 | 76.5 ± 63.2 | 84.5 ± 82.0 | 0.305 |
| Treatment |  |  |  | 0.203 |
| P+T | 89 (25.4%) | 37 (26.2%) | 52 (24.8%) |  |
| TACE | 154 (43.9%) | 68 (48.2%) | 86 (41.0%) |  |
| RT | 108 (30.8%) | 36 (25.5%) | 72 (34.3%) |  |
| P+T: PD-1 inhibitors plus targeted therapy; TACE: Transarterial Chemoembolization; RT: Radiotherapy; AFP: Alpha-Fetoprotein; BCLC: Barcelona Clinic Liver Cancer; PVTT: Portal Vein Tumor Thrombosis; M: Metastasis; AST: Aspartate Aminotransferase | | | | |

**Supplementary Fig. 1.** Decision Curve Analysis of RSF Model at 6 (A), 12 (B), and 24 (C) months. Calibration of RSF Model at 6, 12, and 24 months (D) .


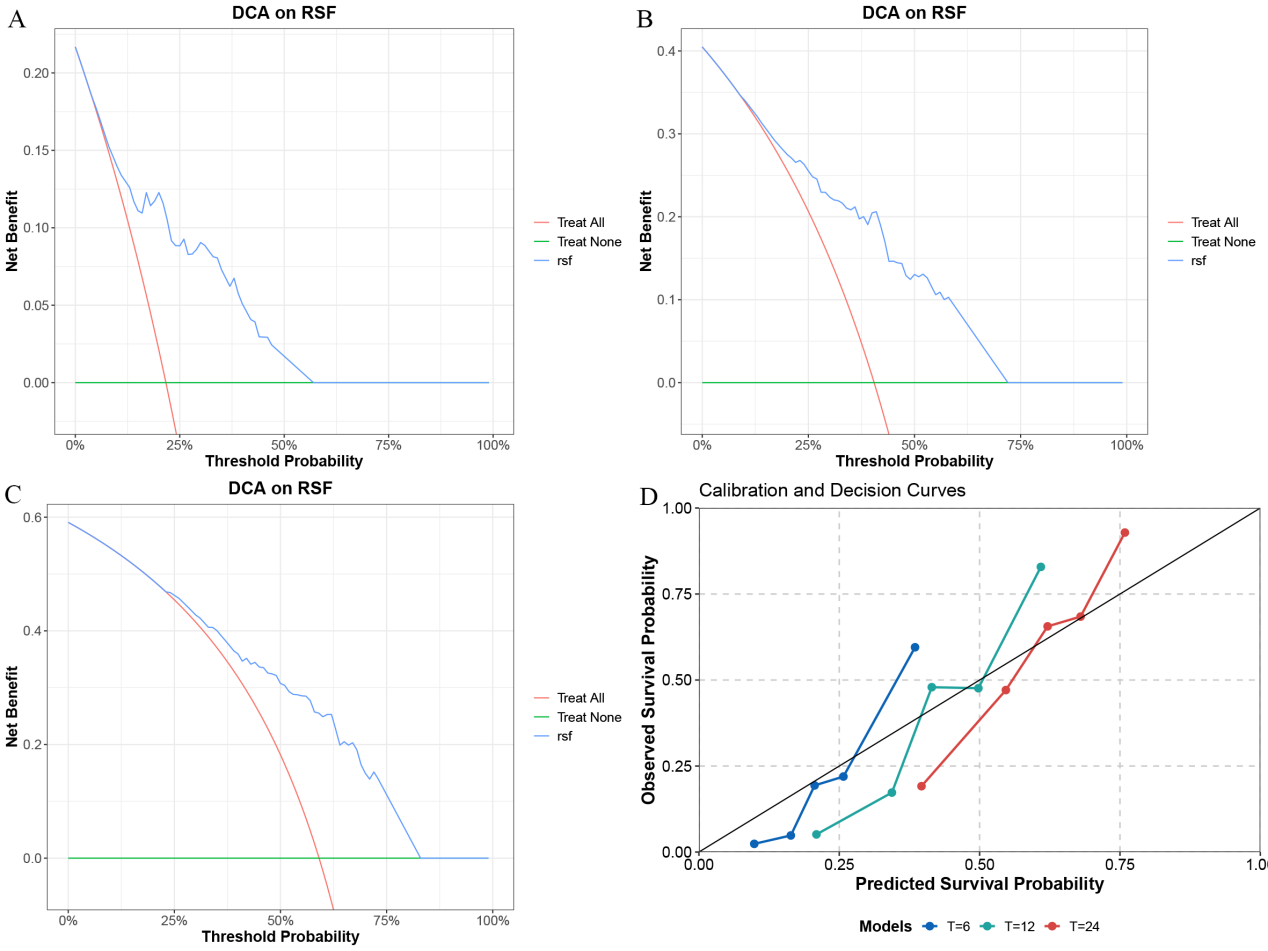

Supplement: Supplementary file 1 [file DataSheet1.docx]
